# *Nasturtium officinale* Microshoot Culture Multiplied in PlantForm Bioreactor—Phytochemical Profiling and Biological Activity

**DOI:** 10.3390/molecules30040936

**Published:** 2025-02-18

**Authors:** Marta Klimek-Szczykutowicz, Magdalena Anna Malinowska, Aleksandra Gałka, Ivica Blažević, Azra Ðulović, Paulina Paprocka, Małgorzata Wrzosek, Agnieszka Szopa

**Affiliations:** 1 Department of Pharmaceutical Sciences, Collegium Medicum, Jan Kochanowski University in Kielce, IX Wieków Kielc 19a, 25-516 Kielce, Poland; 2 Department of Organic Chemistry and Technology, Faculty of Chemical Engineering and Technology, Cracow University of Technology, Warszawska 24, 31-155 Kraków, Poland; magdalena.malinowska@pk.edu.pl (M.A.M.); aleksandra.galka01@gmail.com (A.G.); 3 Department of Organic Chemistry, Faculty of Chemistry and Technology, University of Split, Ruđera Boškovića 35, 21000 Split, Croatia; ivica.blazevic@ktf-split.hr (I.B.); azra.dulovic@ktf-split.hr (A.Ð.); 4 Department of Microbiology and Immunology, Collegium Medicum, Institute of Medical Science, Jan Kochanowski University in Kielce, IX Wiekow Kielc 19A, 25-317, Kielce, Poland; paulina.paprocka@ujk.edu.pl; 5 Department of Biochemistry and Pharmacogenomics, Medical University of Warsaw, Banacha 1, 02-097 Warsaw, Poland; 6 Department of Medicinal Plant and Mushroom Biotechnology, Faculty of Pharmacy, Jagiellonian University Medical College, Medyczna 9, 30-688 Kraków, Poland; a.szopa@uj.edu.pl

**Keywords:** plant in vitro cultures, PlantForm temporary immersion system, antioxidant capacity, anti-elastase activity, anti-tyrosinase activity, anti-microbial activity, cosmetic formula

## Abstract

*Nasturtium officinale* R. Br. (watercress) is an endangered species with valuable pharmaceutical, cosmetic, and nutritional properties. The purpose of this work was to evaluate the phytochemical profile and biological activity of extracts from microshoot cultures grown in PlantForm bioreactors and the parent plant material. After 20 days of cultivation, the cultures achieved the best results both in terms of key active ingredient content and biological activity. The glucosinolates (GSL) profile by the UHPLC-DAD-MS/MS method showed that the dominant compounds were glucobrassicin (493.00 mg/100 g DW, 10 days) and gluconasturtiin (268.04 mg/100 g DW, 20 days). The highest total polyphenol content (TPC) was obtained after a 20-day growth period (2690 mg GAE/100 g DW). Among polyphenols, the dominant compounds in the extracts from in vitro cultures were sinapinic acid (114.83 mg/100 g DW, 10 days) and ferulic acid (87.78 mg/100 g DW, 20 days). The highest antioxidant potential assessed by ABTS and DPPH assays was observed for ethanol extracts. The best results for inhibiting hyperpigmentation (18.12%) were obtained for ethanol extracts and anti-elastase activity (79.78%) for aqueous extract from *N. officinale* microshoot cultures. The extracts from microshoot cultures inhibited the growth of bacteria, including *Cutibacterium acnes* (MIC = 0.625 mg/mL). Antioxidant tests and the chelating capacity of iron ions Fe^2+^ of the face emulsion with *N. officinale* extracts showed higher results than the control.

## 1. Introduction

Plants have the ability to synthesize many chemical compounds, making them one of the main sources of primary and secondary metabolites (SMs) used in food, pharmaceutical, and cosmetic products [1]. The cosmetics industry is increasingly looking for bioactive molecules with beneficial health properties for humans. Currently, there is a growing interest in natural raw materials and substances obtained from them that possess valuable properties for the skin [2]. Metabolites found in plants, especially polyphenolic compounds (phenolic acids, flavonoids) and carotenoids, perform protective functions against, among other factors, UV radiation, drought, and extreme temperatures [3]. In addition, they exhibit antioxidant, anti-aging, anti-microbial, anti-inflammatory, skin whitening, and photoprotective effects on human cells [4,5,6,7,8,9,10,11,12,13,14,15,16].

In the last 10 years, there has been a growing interest in plant cell culture extracts, which contain a wide range of primary and secondary metabolites with many beneficial effects on skin and hair care [4,17,18,19,20,21,22].

*Nasturtium officinale* R. Br. (Robert Brown) belongs to the *Brassicaceae* family, commonly known as watercress. It is a perennial, aquatic, or semiaquatic plant species with creeping or floating stems, which colonizes gently flowing and shallow streams in its natural habitat. This species has been classified by the International Union for Conservation of Nature (IUCN) Red List of Threatened Species as a Plant of Least Concern [23]. The major compounds found in the *N. officinale* herb are glucosinolates (GSLs), isothiocyanates, polyphenols (flavonoids, phenolic acids, proanthocyanidins), terpenoids (including carotenoids), vitamins (B_1_, B_2_, B_3_, B_6_, E, and C), and bioelements [24,25,26,27]. Previous biotechnological studies on in vitro cultures of *N. officinale* have shown their ability to accumulate active compounds such as GSLs and polyphenols in agar and agitated cultures [28], and also in cultures in RITA^®^ bioreactors [29]. Furthermore, it has been shown that extracts from these cultures have antioxidant, anti-inflammatory, and anti-microbial effects [28,29,30,31].

The aim of the research was to further investigate *N. officinale* microshoot cultures to enable large-scale cultivation in the PlantForm TIS and to assess their usefulness by performing phytochemical profiling of GSLs and polyphenolic compounds (PCs) using UHPLC-DAD-MS/MS and HPLC-DAD methods. The biological properties of the extracts were evaluated by testing the antioxidant, anti-elastase, anti-tyrosinase, antibacterial (including anti-acne), and antifungal activities. Additionally, a cosmetic formulation containing a facial care emulsion with the *N. officinale* microshoot culture extract was developed. A series of tests was conducted on the obtained formulation, including an evaluation of its physicochemical properties and its biological activity.

## 2. Results

### 2.1. Individual GSL Contents

The qualitative and quantitative analysis using UHPLC-DAD-MS/MS confirmed the presence of four GSLs (7-(methylsulfinyl)heptyl GSL, glucohirsutin, gluconasturtiin, and glucobrassicin) in *N. officinale* microshoots extracts after 10 days of cultivation and three GSLs (7-(methylsulfinyl)heptyl GSL, glucohirsutin, and gluconasturtiin) after 20 days of cultivation (Table 1). Notably, glucobrassicin was detected in high amounts (493.00 mg/100 g dry weight (DW)) after 10 days but was absent after 20 days of growth. In vitro cultures (Figure S1) also showed high gluconasturtiin contents, ranging from 207.91 mg/100 g DW (10 days of growth period) to 268.04 mg/100 g DW (20 days of growth period) (Table 1).

### 2.2. Total Phenolic Assay and Polyphenol Profiling by HPLC-DAD

The total polyphenol content (TPC) in the obtained plant extracts was determined using the Folin–Ciocalteau (FC) assay (Table 2). In vitro cultures were characterized by a similar concentration of polyphenolic compounds (PCs) as the plant material. The highest concentration was obtained after 20 days and was 2690 mg equivalent of gallic acid (GAE)/100 g DW. However, in the extracts from the parent plant, the concentration was 2940 mg GAE/100 g DW.

According to our former study [29] using the UHPLC-DAD-MS/MS method, we confirmed the presence of individual phenolic compounds in *N. officinale* agar microshoot extracts. Studies have shown the presence of three phenolic acids (ferulic acid, protocatechuic acid, and sinapinic acid) and one flavonoid (rutoside), the content of which was assessed using the HPLC-DAD method.

Quantitative analysis showed that the dominant compound in the extracts from in vitro cultures was sinapinic acid; its content was dependent on the growth cycles and was estimated at 114.83 and 78.65 mg/100 g DW after 10 and 20 days of growth period, respectively (Table 2). The content of ferulic acid was also high similar at 10 (57.99 mg/100 g DW) and 20 days (87.78 mg/100 g DW) of growth period (Table 2). Among the phenolic acids, the lowest contents were obtained for protocatechuic acid—8.75 mg/100 g DW (after 10 days) and 15.65 mg/100 g DW (after 20 days). The amount of rutoside was calculated as 13.40 and 33.27 mg/100 g DW after 10 and 20 days of growth period, respectively (Table 2).

### 2.3. Assessment of Biological Activity

The extracts used in the antioxidant activity assays as well as enzymatic tests were applied at a concentration of 125 µg/mL. The same concentration was used for the reference substances to enable a comparison of the obtained activity values.

#### 2.3.1. Total Antioxidant Potential Assay by ABTS

**Figure 1:**
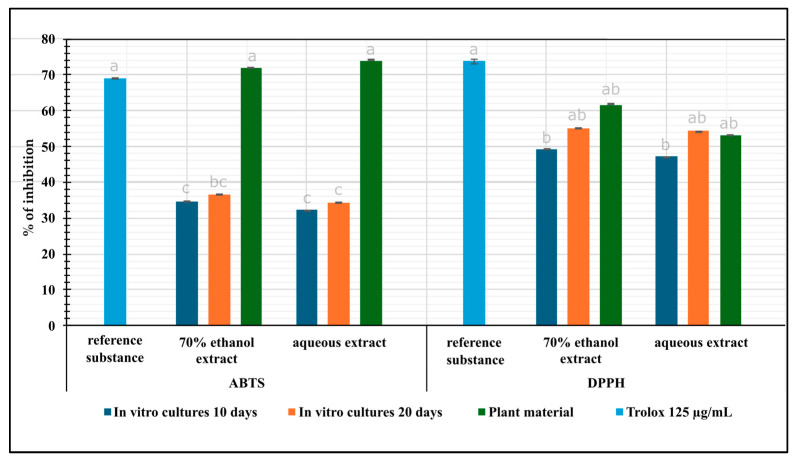
Antioxidant potential, estimated by the ABTS and DPPH assays (% inhibition ± SD), of *N. officinale* extracts from the herb and microshoots cultivated in the PlantForm bioreactor (extract concentrations: 125 µg/mL).

The three assays—ABTS (2,2′-azino-bis(3-ethylbenzothiazoline-6-sulfonic acid), DPPH (2,2-diphenyl-1-picrylhydrazyl), and chelating ability—were used to estimate the antioxidant potential of *N. officinale* microshoot bioreactor extracts. For comparative purposes, an analysis of extracts from the herb of the parent plant was performed (Figure 1). The antioxidant potential of biomass extracts for in vitro cultures analyzed by ABTS assay, ranged from 32.23% for the aqueous extract of cultures after 10 days to 36.69% for the ethanolic extract of cultures after 20 days. Inhibition of extracts from the parent plant herb was 71.92% (Figure 1).

#### 2.3.2. Total Antioxidant Activity Assay by DPPH

The antioxidant potential of experimental biomass extracts estimated with the DPPH method ranged from 47.15% to 55.22% and the highest activity was determined for the ethanolic extracts after 20 days of cultivation. For extracts from the parent plant material, inhibition was 61.45% (Figure 1).

#### 2.3.3. The Chelating Capacity of Iron Ions Fe^2+^

**Figure 2:**
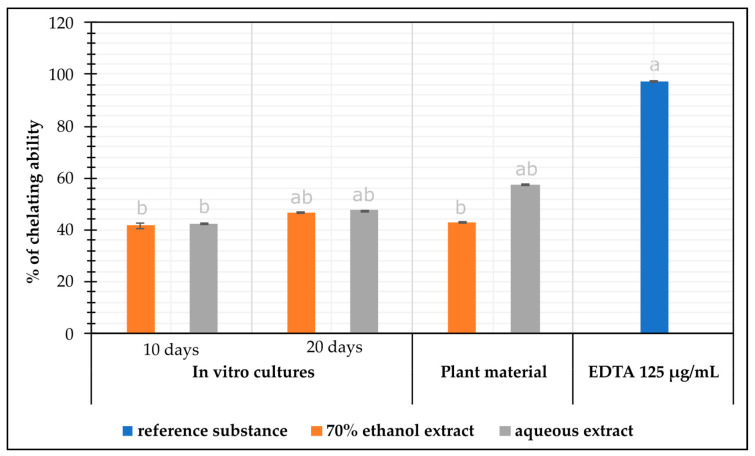
The chelating capacity of iron ions Fe^2+^ (% chelating ability±SD) of *N. officinale* extracts from the herb and microshoots cultivated in the PlantForm bioreactor (extract concentrations: 125 µg/mL).

In the analysis of the ability to chelate iron (II) ions, the average percentage of inhibition for in vitro extracts ranged from 41.79% (ethanolic extract, 10 days) to 47.50% (aqueous extract, 20 days). Comparing the extracts obtained from in vivo plants, it can be seen that the extracts showed a similar chelating ability to in vitro cultures (43.08%) (Figure 2).

#### 2.3.4. Elastase Inhibition Ability Assay

**Figure 3:**
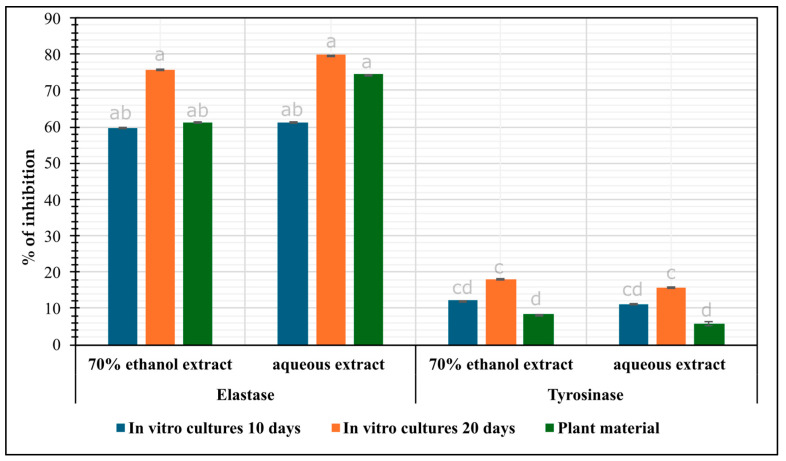
Elastase and tyrosinase inhibition ability (% inhibition ± SD) of *N. officinale* extracts from the herb and microshoots cultivated in the PlantForm bioreactor (extract concentrations: 125 µg/mL).

Studies conducted to evaluate elastase inhibition showed greater inhibition capacity by in vitro culture extracts (Figure 3). The elastase inhibition capacity ranged from 75.88% (ethanolic extract, 10 days) to 79.78% (aqueous extract, 20 days). Extracts from the parent herb showed elastase inhibition ranging from 61.30% (ethanolic extract) to 74.45% (aqueous extract) (Figure 3).

#### 2.3.5. Tyrosinase Inhibition Ability Assay

Figure 3 presents the results of the study on tyrosinase inhibition activity, which is directly related to skin-lightening effects. The best results for inhibiting hyperpigmentation were obtained with the ethanolic extract after 20 days of cultivation (18.12%), while a slightly weaker effect was observed with the aqueous extract after 10 days of cultivation (11.09%) (Figure 3). The lowest activity was shown by the extract obtained from the parent plant (5.87–8.32%) (Figure 3).

#### 2.3.6. Anti-Microbial Assay

The minimal inhibitory concentration (MIC) determined for the extracts of in vitro *N. officinale* cultures ranged from 0.625 to 1.25 mg/mL (Table 3). For the tested Gram-positive bacterial strains the values differed, ranging from 0.625 mg/mL (*C. acnes*) to 1.25 mg/mL (*S. aureus* and *S. epidermidis*) The MIC for Gram-negative bacteria (*E. coli*) was 1.25 mg/mL. The minimum bactericidal concentration (MBC) of the in vitro culture extracts was estimated at 0.625–5 mg/mL. Among the tested bacterial strains, the highest MBC (5 mg/mL) was noted for *S. aureus*, while the lowest (0.625 mg/mL) was detected for *C. acnes* (Table 3). For bioreactor microshoot culture extracts, the MIC for the tested fungal strain—*C. albicans*—equal 1.25 mg/mL (Table 3).

In *N. officinale* herb extract [29] the MIC was equal to 5 mg/mL for *S. aureus*, and 10 mg/mL for *S. epidermidis* and *E. coli*. Additionally, for *C. albicans* the herb extracts, MIC was 5 mg/L. The studies showed a higher potential of microshoot *N. officinale* bioreactor cultures compared to parent plant material [29].

### 2.4. Study on Cosmetic Formulation

#### 2.4.1. Physicochemical Tests

##### Stability Tests and pH Measurement

The stability tests confirmed the stability of the preparations, as both samples of cosmetic emulsions tested did not separate. In the variable temperature test, for the emulsion without *N. officinale* extract, no separation of the sample was observed. The measured pH value differed slightly between the prepared emulsions. The average pH values of the emulsions ranged from 6.21 to 6.25. According to the data in the literature, cosmetic preparations should have a pH close to that of human skin, i.e., slightly acidic, in the range of 4 to 6 [32]. The results obtained for both emulsions are within the required pH range; however, in the emulsion with the addition of the plant extract, the pH value was lower (6.25 for the emulsion without extract and 6.21 for the emulsion with extract, respectively).

##### Rheological Study of Emulsions

The results for the samples obtained from the rheological measurements were analyzed and averaged. Then, the viscosity was plotted. Table 4 presents a comparison of the viscosity values of the preparations at selected shear rate values.

According to the values presented in Table 4, the highest overall shear viscosity was obtained for the emulsion with the extract. The findings suggest that the extract could be a valuable functional ingredient in cosmetic products aimed at safeguarding skin health against environmental stressors.

The higher viscosity of the emulsion with the *N. officinale* extract is likely due to the presence of bioactive compounds, such as polyphenols, glucosinolates, or other secondary metabolites, which can interact with the water and oil phases of the emulsion. These interactions may result in increased hydrogen bonding, network formation, or the thickening effect of polysaccharides present in the extract [32]. Additionally, the extract may influence the structural arrangement of the emulsion’s internal phase, leading to enhanced resistance to shear forces and, consequently, higher viscosity.

#### 2.4.2. The Total Antioxidant and Chelating Ability of the Emulsions

**Figure 4:**
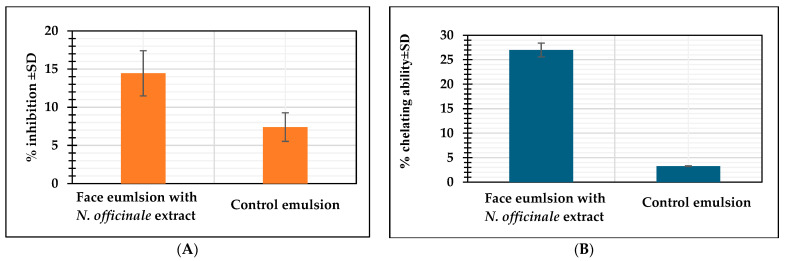
Antioxidant potential, DPPH assay (% inhibition ± SD) (**A**), and the chelating capacity of iron ions Fe^2+^ (% chelating ability ± SD) (**B**) of tested emulsions.

The results of the total antioxidant capacity and chelating properties of the prepared emulsions are presented in Figure 4. These findings pertain to the biological activity of the final formulations, allowing for an assessment of their actual effects on the skin. A comparison was made between the effects of the emulsion without extract and the emulsion containing 2% *Nasturtium officinale* extract. 

The results obtained reveal notable differences in antioxidant activity and chelating ability between emulsions containing *N. officinale* extract and those without it. The emulsion with 2% *N. officinale* extract exhibited significantly higher total antioxidant activity, averaging 14.45%, compared to 7.40% in the control emulsion without the extract. This twofold increase in antioxidant activity demonstrates the potent contribution of *N. officinale* extract in scavenging free radicals, which is essential for formulations aimed at reducing oxidative stress on the skin.

Similarly, the chelating ability of the emulsion containing *N. officinale* extract was markedly higher, reaching 26.98%, while the control emulsion showed only 3.27%. The chelation of metal ions, such as iron, is crucial in skincare due to its role in inhibiting oxidative reactions that can damage cellular structures and accelerate skin aging. The significantly greater chelating ability observed in the emulsion with *N. officinale* indicates its added protective potential against oxidative processes driven by metal ions.

## 3. Discussion

The studies carried out were aimed at assessing extracts from *N. officinale* microshoot cultures maintained in the PlantForm bioreactor with regard to phytochemical studies and analysis of biological activities, and their potential future use in cosmetic products. In the studies, the production of GSLs and polyphenols was analyzed. Additionally, we conducted studies on potential biological activities, including antioxidant potential, anti-elastase, anti-tyrosinase, and anti-microbial properties. The results were compared with those obtained for extracts of the parent plant [29]. Additionally, physicochemical properties of a cosmetic emulsion containing *N. officinale* extract were investigated and compared to an emulsion without the extract.

The study confirmed variation in GSL content depending on the growth period (Table 1). In the PlantForm bioreactor, the content of gluconasturtiin was 1.3-fold higher after 20 days of growth period compared to 10 days (Table 1). Gluconasturtiin, the major GSL occurring in *N. officinale* herb, reached 640.94 mg/100 g DW in extracts of the herb [29], which was 2.4 fold higher than in extracts of *N. officinale* grown in the PlantForm bioreactor after 20 days of growth (Table 1). The herb extracts were also shown to contain methionine-derived GSLs (7-(methylsulfinyl)heptyl GSL, 92.27 mg/100 g DW, and glucohirsutin, 42.79 mg/100 g DW), and tryptophan-derived GSLs (4-methoxyglucobrassicin, 23.47 mg/100 g DW) [29], which occurred in trace amounts or were not detected in extracts of microshoot cultures grown in PlantForm bioreactor. Interestingly, a high content of glucobrassicin was obtained after 10 days of cultivation, whereas it was absent in bioreactor cultures after 20 days of cultivation, and present in trace amounts in the herb of the parent plant (Table 1). Additionally, the herb of the parent plant contained glucohesperin, 7-(methylsulfanyl)heptyl GSL, and 8-(methylsulfanyl)octyl GSL in traces, while they were not present in the *N. officinale* PlantForm bioreactor microshoot cultures [29]. Jeon et al. reported the highest gluconasturtiin content in flowers (7.39 mg/100 g DW), which was about 36.3-fold lower than in extracts of *N. officinale* PlantForm bioreactor microshoot cultures after 10 days of growth period [27]. Another study conducted by Giallourou et al. [33], using the LC-MS/MS technique identified five GSLs: gluconasturtiin, glucobrassicin, 4-hydroxyglucobrassicin, 4-methoxyglucobrassicin, and 7-(methylsulfinyl)heptyl GSL. The main compound detected in their study was gluconasturtiin (176.00 mg/100 g DW), which was 1.2-fold lower than in our extracts from herb and was comparable to 10-day extracts of PlantForm bioreactor microshoot cultures. Rubin et al. [34] investigated qualitative and quantitative GSL content in extracts from agar shoot cultures grown for 30 days on MS agar medium without plant growth regulators. The amount of gluconasturtiin (140.40 mg/100 g FW) determined in their study was 1.5-fold lower than in PlantForm bioreactor microshoot cultures harvested after 10 days of growth [34]. Studies show that bioreactor cultures conducted in TIS systems promote biomass growth and better aeration of cultures, and due to constant growth conditions, as well as the availability of nutrients and light, etc.; this contributes to greater production of active ingredients. Other studies conducted on various plant species grown in these systems, such as *Plumeria rubra*, *Schisandra henryi*, and *Pontechium maculatum*, also confirmed the positive effect on the accumulation of secondary metabolites in these cultures [35,36,37]. The higher TPC was obtained for extracts from *N. officinale* herb (2940 mg GAE/100 g DW) and it was 1.1 times and 1.2 times higher than in extracts from microshoot cultures after 20 and 10 days of growth period, respectively (Table 2). In extracts from *N. officinale* microshoot cultures gown in RITA^®^ bioreactor (VITROPIC, Saint-Mathieu-de-Tréviers, France), TPC was higher in extracts from cultures grown for 10 days than in extracts from the herb and grown in the bioreactor for 20 days, which indicates differences in the compound content due to the type of bioreactor used [29]. TPC of other species of the Brassicaceae family showed lower contents compared to extracts of *N. officinale*. In the extracts of *Eruca sativa* leaves, the TPC was 8.13 mg GAE/100 g DW, while, in *Brassica oleracea var. italica*, it was 8.32 mg/100 g DW, which was 361.6 and 353.4 times lower than in extracts of *N. officinale* herb (Table 2) [38]. The TPC in extracts from aerial parts of *Lepidum sativum* was 10.96 mg GAE/g DW, and it was 2.7 times lower than in extracts of *N. officinale* herb (Table 2) [39]. Arena et al. conducted a study on extracts from the leaves of *Sinapis pubescens* subsp. *pubescens*, which showed that the TPC was 64.06 mg GAE/g DW, which was 2.2 times higher than in extracts of *N. officinale* herb (Table 2) [40].

Quantitative differences in the contents of phenolic acids and flavonoids were confirmed depending on the duration of *N. officinale* PlantForm bioreactor microshoot cultures cultivation (Table 2). The highest contents for ferulic acid, protocatechuic acid, and rutoside were obtained after 20 days of growth, and these were 1.5, 1.8, and 2.5 times higher than after 10 days, respectively. Only the content of sinapinic acid was about 1.5 times higher after 10 than after 20 days. The extracts of the *N. officinale* herb [29] showed the presence of the following phenolic acids: *p*-coumaric, ferulic, gallic, sinapinic, and protocatechuic acids. Among flavonoids, isoquercitrin, kaempferol *O*-rhamnohexoside, and rutoside were detected. Both qualitative and quantitative differences are visible in the microshoot cultures grown in the PlantForm bioreactor compared to the results of the extracts from the *N. officinale* herb. In herb *N. officinale* the main phenolic acid found was protocatechuic acid, whose content was 12.5-fold higher than in PlantForm bioreactor microshoot cultures (Table 2). However, in *N. officinale* microshoot cultures grown in the PlantForm bioreactor, the amount of sinapinic acid was 8.8 times higher (Table 2). Among flavonoids, the presence of rutoside was confirmed in the extracts from bioreactor microshoot cultures, and this was 4.6-fold higher than in *N. officinale* herb (Table 2) [29].

The phenolic acids and flavonoids content were analyzed using the UHPLC-DAD-ESI-MS and HPLC-DAD methods in extracts from *N. officianle* microshoot cultures grown on a variant MS medium containing 1 mg/L BA and 1 mg/L NAA in the TIS RITA^®^ bioreactor [29]. In extracts of *N. officianale* microshoot cultures grown in the RITA^®^ bioreactor, the main phenolic acid was protocatechuic acid, whose amount was 9.8 times higher compared to extracts from 10-day microshoot cultures of the PlantForm bioreactor. However, in microshoot cultures from the Plantform bioreactor, the content of sinapinic acid was obtained 20.3 times higher (Table 2). The highest content of rutoside was observed in both bioreactors after 20 days of growth, and it was 1.4 times higher in extracts from the PlantForm bioreactor (Table 2) [29]. These studies confirm that the selection of the bioreactor and the duration of biomass immersion in the medium may also affect the content of active ingredients in in vitro cultures.

The antioxidant potential analyzed using ABTS and DPPH assays demonstrated the high potential of PlantForm TIS-grown microshoot and herb extracts (Figure 1).

In the ABTS assay, the highest antioxidant potential was obtained for aqueous extracts of the herb, which was approximately 2.3-fold and 2.2-fold higher than in extracts from microshoot cultures grown for 10 and 20 days, respectively (Figure 1). The antioxidant potential was similar in ethanolic and aqueous extracts, and there were no major differences between the days of in vitro culture growth. In the DPPH assay, among all extracts, both for the herb and for in vitro cultures, the highest antioxidant potential was obtained for ethanolic extracts. The antioxidant potential of ethanol extracts from the herb was about 1.2-fold and 1.1-fold higher than in extracts from microshoot cultures grown for 10 and 20 days, respectively (Figure 1). In extracts of *N. officinale* RITA^®^ bioreactor microshoot cultures [29], a higher antioxidant potential of herb extracts was also observed compared to microshoot cultures. Interestingly, in this study, a large difference was observed between herb extracts and PlantForm bioreactor microshoot cultures only for the ABTS assay (Figure 1). However, in the DPPH assay, these differences were not large. Moreover, among PlantForm bioreactor microshoot cultures, the highest antioxidant potential in the DPPH assay was obtained for cultures grown for 20 days, while for cultures grown in the RITA^®^ bioreactors, the highest potential for the DPPH method was obtained after 20 days (Figure 1) [29]. Sharafan et al. [17] conducted antioxidant potential studies on extracts from agar in vitro cultures of different cultivars of *Vitis vinifera*. The studies showed that in the DPPH assay, the highest inhibition (33.57%) was observed for *V. vinifera* cvs. Johanniter, which was lower than in the extracts obtained by us from in vitro cultures of *N. officinale* (55.22%) (Figure 1).

In the analysis of the ability to chelate iron (II) ions, the maximum potential was obtained for aqueous extracts of the herb. The maximum potential of the herb extracts was approximately 1.4-fold and 1.2-fold higher than in extracts from microshoot cultures grown for 10 and 20 days, respectively (Figure 2).

The studies confirm for the first time the elastase-inhibition properties of *N. officinale* microshoot extracts. The highest inhibition was obtained for aqueous extracts. The strongest inhibition was demonstrated by aqueous extracts from 20-day PlantForm bioreactor microshoot cultures (79.78%) and was 1.1 and 1.3 times higher than in extracts from herb and extracts from 10-day PlantForm bioreactor microshoot cultures, respectively (Figure 3). Mansinhos et al. [41] conducted studies on extracts from agar in vitro cultures of *Lavandula viridis* and *Thymus lotocephalus*. The studies showed that extracts from in vitro cultures of *L. viridis* inhibited elastase by 20.48%, while extracts from in vitro cultures of *T. lotocephalus* inhibited elastase by 23.00%, which was a weaker result than for *N. officinale* (Figure 3). Bose et al. [42], in their studies on leaf, stem, and in vitro culture extracts of *Musa acuminata* showed a strong elastase-inhibiting effect for all extracts tested. This study confirmed that extracts from in vitro cultures were characterized by lower inhibitory activity than parent plant material. The optimal conditions and the use of bioreactor cultures, it is possible to obtain microshoot cultures that show higher elastase inhibition than plant material. Studies have shown that species from the Brassicaceae family may have potential use in anti-aging preparations. Szewczyk et al. [43] conducted studies on elastase inhibition of extracts from *Eutrema japonicum*. The highest activity was demonstrated by the extract from the root of *E. japonicum* (90.18%).

In order to investigate the skin-lightening potential, tyrosinase inhibitory activity was also tested. The highest inhibition was obtained for ethanolic extracts (Figure 3). In addition, the highest inhibitory effect was obtained for extracts from 20-day PlantForm bioreactor microshoot cultures (18.12%), which was approximately 2.2-fold and 1.5-fold higher than in extracts from the herb and extracts from 10-day PlantForm bioreactor microshoot cultures, respectively (Figure 3). The studies on the inhibition potential of agitated microshoot cultures treated with 100 μM methyl jasmonate (MeJA) and collected after 24 h of elicitation showed that extracts from elicited cultures inhibited tyrosinase in 27.84%, which was 1.5-fold higher inhibition capacity of PlantForm grown tissue [31]. In the studies of Mansinhos et al. [41], extracts from in vitro agar cultures of *L. viridis* and *T. lotocephalus* showed higher inhibitory activity on tyrosinase at 39.35% and 28.30%, respectively. This was 2.2 and 1.6 times higher than in 20-day PlantForm bioreactor microshoot cultures, respectively (Figure 3). Sharafan et al. [17] conducted studies on elastase inhibition by extracts from in vitro agar cultures of different cultivars of *Vitis vinifera*. The studies showed that the highest inhibition (17.50%) was obtained for *V. vinifera* cvs. Hibernal and it was similar to that obtained from in vitro extracts of *N. officinale* (18.12%) (Figure 4).

The studies assessed the antibacterial activity against Gram-positive bacteria: *S. aureus*, *S. epidermidis*, *C. acnes*, and Gram-negative: *E. coli* and the antifungal activity against *C. albicans*. The anti-microbial analysis for extract from *N. officinale* microshoots grown in RITA^®^ bioreactors and herb of parent plant showed that extracts from PlantForm bioreactors microshoot cultures showed 4-fold lower MIC for *S. aureus* and *S. epidermidis* and 8-fold lower MIC for *E. coli* [29]. Extracts from PlantForm bioreactor microshoot cultures also demonstrated a lower MIC for *C. albicans* by 8-fold and 4-fold compared to extracts from RITA^®^ bioreactor microshoot cultures and herb of the parent plant, respectively [29]. Additionally, previous studies [44] demonstrated the antibacterial activity of extracts from *N. officinale* herb toward *E. coli*, *Klebsiella pneumoniae*, *Enterococcus faecalis*, and *Bacillus cereus*. In the study of Quezada-Lazaro et al. [45], the anti-microbial activity of the herb extracts against *Mycobacterium tuberculosis* was confirmed. In the present study, we confirmed the antibacterial and antifungal potential of these extracts, especially against skin pathogens. The lowest MIC was obtained for *C. acnes*, which may indicate its potential use of extracts from PlantForm bioreactor microshoot cultures in anti-acne products.

Differences in biological activity may result from the varying contents of compounds obtained in in vitro cultures, as well as in plant material. As can be seen in Table 1 and Table 2, in in vitro cultures, other compounds predominate in plant material, which may indicate effectiveness against a given radical, enzyme, bacteria, or fungus. In biological activity studies, 20-day in vitro cultures had greater enzyme inhibition capabilities. Based on the analysis of compound contents, it can be suggested that this effect of extracts was influenced by polyphenolic compounds, which were present in higher concentrations in these extracts. Moreover, the much higher content of glucobrassicin in 10-day cultures did not significantly enhance the potential biological activity of the extracts. Additionally, the higher GSL contents in herb extracts may have contributed to the comparatively higher results observed in the antioxidant potential study.

The promising results observed in the phytochemical profiling, along with antioxidant, anti-aging, and skin-brightening biological activities, motivated the development of a cosmetic formulation incorporating an emulsion with an extract derived from experimental microshoots cultivated in Plantform bioreactors. Both the formulation containing the extract and a control formulation without the extract underwent technical parameter assessments, which demonstrated stability and favorable rheological properties. The obtained emulsions were stable and exhibited pH values suitable for the skin (6.21–6.23). The pH value of cosmetic products plays a critical role in maintaining skin health and integrity [46].

The emulsion produced in this study is classified as an oil-in-water (O/W) type, as indicated by its physical properties and composition. This type of emulsion is particularly suitable for cosmetic and dermatological applications, as it is easily absorbed by the skin and provides a hydrating effect [47].

The viscosity curves of the emulsions showed minimal variation between formulations, demonstrating consistent flow properties. However, slight differences in the tangential stress values were observed at the same shear rates, indicating subtle variations in the internal structure of the emulsions. Additionally, the flow curves for the emulsions were similar, suggesting comparable rheological profiles across the samples. Notably, the inclusion of the extract in the emulsions led to a slight increase in viscosity, which positively impacted the sensory properties of the emulsions, improving the perceived consistency and texture. This enhanced viscosity is beneficial for both product stability and user experience [48,49].

Additionally, face emulsion with *N. officinale* extracts confirmed about 2.0-fold and 8.3-fold higher antioxidant potential and chelating ability of Fe^2+^ ions, respectively, than control emulsion (Figure 4B). These results confirm the efficacy of *N. officinale* extract in enhancing the antioxidant and metal-chelating properties of skincare emulsions, supporting its role in formulations targeting skin protection, anti-aging, and rejuvenation. The findings suggest that the extract could be a valuable functional ingredient in cosmetic products aimed at safeguarding skin health against environmental stressors.

## 4. Materials and Methods

### 4.1. Experimental In Vitro Cultures

Initial microshoot cultures of *N. officinale* were established from seeds, which were obtained from the Garden of Medicinal Plants, Jagiellonian University, Medical College, Faculty of Pharmacy, Cracow, Poland. A voucher specimen (No. 102) has been deposited in the herbarium of this Garden. The methodology for initiation has been reported previously [49]. The seeds were surface-sterilized in 0.1% mercuric chloride solution (HgCl_2_) for 5 min. The seeds were continuously stirred in this solution and then washed three times with sterile distilled water. Subsequently, the sterilized seeds were inoculated to standard Murashige and Skoog (MS) medium [50] with 3% (*w*/*v*) sucrose and supplemented with 1 mg/L BA (6-benzyladenine) and 1 mg/L NAA (1-naphthaleneacetic acid). Experimental microshoots were cultivated in PlantForm TIS bioreactors (PlantForm MWb & AJS, Hjarup, Sweden) (Figure S1A,B) containing 500 mL of MS medium supplemented with 1 mg/L BA and 1 mg/L NAA. The medium was identified as optimal for the cultivation of microshoots in the previous studies [28]. The inoculum used for one PlantForm vessel was composed of 10 g FW (fresh weight) of *N. officinale* microshoots. The experimental media and biomass samples were collected after growth periods of 10 and 20 days (three series). The microshoots were grown under continuous exposure to an LED white light (2.75 W/m^2^) at a temperature of 25 ± 2 °C. The immersion cycle was set to 5 min every 1.5 h, at an aeration rate of 1.0 vvm. The material for analyses was lyophilized (Labconco Corporation, Kansas City, MO, USA) prior to analysis. The morphological appearance of the in vitro cultures and plant material (*N. officinale* herb) as well as the results regarding the biomass growth parameters were included in the Supplementary Data (Figure S1).

### 4.2. Parent Plant Material

The *Nasturtium officinale* R. Br. plant material was obtained from the Garden of Medicinal Plants, Faculty of Pharmacy, Jagiellonian University, Medical College, Cracow, Poland, loc. 50.01° N, 19.99° E, in May 2018. Voucher specimen (No. 102) has been deposited in the herbarium of this Garden. The material for analyses was lyophilized (Labconco Corporation, Kansas City, MO, USA) prior to analysis.

### 4.3. Extraction

For phytochemical and anti-microbial studies, the pulverized biomass obtained from in vitro cultures was weighed (0.2 g) and extracted twice in 4 mL of methanol (STANLAB, Lublin, Poland) under sonication for 30 min (ultrasonic bath; POLSONIC 2, Warsaw, Poland). The extracts were centrifuged (7 min, 2000× *g*; MPW-223E; MPW, Warsaw, Poland) and filtered (0.22 μm syringe filters; Millex^®^ GP; Merck Millipore, Burlington, MA, USA).

For antioxidant, anti-elastase, and anti-tyrosinase potential assessment, two types of extracts—aqueous and ethanolic—were prepared as follows. *N. officinale* extracts from microshoots cultures and herbs were obtained by measuring 0.25 g lyophilized biomass and adding 2.5 mL of deionized water or 70% ethanol (STANLAB, Lublin, Poland). Then, the samples were extracted using an ultrasound-assisted extraction method for 30 min (ultrasonic bath, IS-3 by InterSonic (Olsztyn, Poland)). The extracts were centrifuged (15 min, 35,000 rpm, EBA 20 by Hettich (Beverly, MA, USA)) to separate the extract from the sediment.

### 4.4. Analysis of GSLs

Desulfoglucosinolates (dGSLs) were isolated from 100 mg of dried plant material as reported previously [29,51,52]. The standard used, sinigrin, was obtained from Sigma-Aldrich (St. Louis, MO, USA); glucohesperin (1), glucohirsutin (3), gluconasturtiin (6), glucobrassicin (7), and 4-methoxyglucobrassicin (8) were obtained from Phytoplan (Heidelberg, Germany). First, the plant material was extracted with a methanol/H_2_O mixture (70:30, *v*/*v*; Gram-Mol d.o.o., Zagreb, Croatia). The resulting supernatant was loaded onto a mini-column filled with DEAE-Sephadex A-25 anion-exchange resin (Sigma-Aldrich, St. Louis, MO, USA). The remaining nonpolar compounds were removed by washing the columns. To create appropriate conditions for the sulfatase reaction, the mini-columns were washed again with 20 mM NaOAc buffer (Merck, Darmstadt, Germany), followed by the addition of sulfatase (type H-1 from Helix pomatia; Sigma-Aldrich, St. Louis, MO, USA). The column was left overnight to allow the reaction to occur, and dGSLs were eluted the next day with ultrapure H_2_O (Merck Millipore, Burlington, MA, USA).

The analysis of extracts was performed by a UHPLC-DAD-MS/MS apparatus (Ultimate 3000RS with TSQ Quantis MS/MS detector; Thermo Fischer Scientific, Waltham, MA, USA) using a Hypersil GOLD column (3.0 µm, 3.0 × 100 mm; Thermo Fischer Scientific, Waltham, MA, USA). A gradient consisting of solvent A (50 μM NaCl in H_2_O) and solvent B (acetonitrile:H_2_O, 30:70, *v*/*v*) was applied at a flow rate of 0.5 mL/min as follows: 0.14 min, 96% A, and 4% B; 7.84 min, 14% A, and 86% B; 8.96 min, 14% A, and 86% B; 9.52 min, 5% A and 95% B; 13.16 min, 5% A and 95% B; 13.44 min, 96% A, and 4% B; as well as 15.68 min, 96% A and 4% B. The column temperature was held at 25 °C, and the injection volume was 5 µL. The system was operated in the positive ion electrospray mode, with the electrospray interface H-ESI operating at a capillary voltage of 3.5 kV and a temperature of 350 °C. The signals were recorded at 227 nm by a DAD. dGSLs were quantified using an external calibration curve of pure desulfosinigrin (range from 13.56 to 542.50 µM). For each individual dGSL, response factors (RPFs) were taken into account in accordance with the literature as follows: RPF 1.0 for glucohesperin, 1.1 for glucohirsutin and 8-(methylsulfanyl)octyl GSL [53], 0.95 for gluconasturtiin, 0.29 for glucobrassicin, and 0.25 for 4-methoxyglucobrassicin [54]; arbitrary 1.0 for 7-(methylsulfinyl)heptyl GSL and 7-(methylsulfanyl)heptyl GSL.

### 4.5. Total Phenolic Assay

The F-C assay described by Singleton and Rossi [55] was used to measure the TPC in the plant extract samples. The absorbance measurement was performed at a wavelength of 765 nm using a multiwell plate reader (Infinite M Nano multiplate reader, Tecan, Männedorf, Sweden). The standard curve prepared for the appropriate concentrations of gallic acid was used to determine the polyphenol content.

### 4.6. Polyphenol Profiling by HPLC-DAD

This analysis was performed using the HPLC-DAD method according to the method described in previous works [56,57]. Methanolic extracts (prepared as described in Section 4.4) were used. An HPLC-DAD system (Merck-Hitachi, LaChrom Elite, Darmstadt, Germany) with a DAD L-2455 detector. Separation was performed on a Purospher RP-18 analytical column (4 × 250 mm, 5 μm; Merck, Boston, MA, USA) was used for the analysis. Elution was performed with a mobile phase A (methanol:0.5% acetic acid, 1:4, *v*/*v*) and a mobile phase B (methanol). The gradient program was set as follows: 0–20 min, 0% B; 20–35 min, 0–20% B; 35–45 min, 20–30% B; 45–55 min, 30–40% B; 55–60 min, 40–50% B; 60–65 min, 50–75% B; and 65–70 min, 75–100% B. The hold time was 15 min. The other parameters were as follows: temperature, 25 °C; flow rate, 1 mL/min; injection volume, 10 μL; and detection wavelength, 254 nm. Quantitative analysis was carried out for the following compounds detected and confirmed in the extracts using UHPLC-DAD-ESI-MS: *p*-coumaric acid, ferulic acid, gallic acid protocatechuic acid, and rutoside (all standard compounds were purchased from Sigma-Aldrich Co., St. Louis, MO, USA).

### 4.7. Assessment of Biological Activity

#### 4.7.1. Total Antioxidant Potential Assay by ABTS

The antioxidant potential of the extracts obtained from the tested biomass was estimated using the ABTS assay [58]. The absorbance measurement was performed using a microplate reader at a wavelength set at 630 nm using multiplate reader (Infinite M Nano multiplate reader, Tecan, Männedorf, Sweden). Reference substance was trolox 125 µg/mL. The total antioxidant activity was expressed as the percentage of oxidation process inhibition relative to the control sample without the extract.

#### 4.7.2. Total Antioxidant Potential Assay by DPPH

The free radical-scavenging activity of the extracts was determined using the stable radical DPPH assay, as described by Brand-Williams [59]. The measurement was performed using 96-well plates. Absorbance was measured at a wavelength of 517 nm (Infinite M Nano multiplate reader, Tecan, Männedorf, Sweden). Reference substance was trolox at a concentration of 125 µg/mL. The total antioxidant activity was determined as the percentage reduction in oxidation processes compared to a control without the extract.

#### 4.7.3. The Chelating Capacity of Iron Ions Fe^2+^

The iron (II)-chelating capacity of extracts from *N. officinale* microshoot cultures and the parent plant herb was investigated, using the method described by Adjimani and Asare [60]. Absorbance was measured at a wavelength of 562 nm using the multiplate reader (Infinite M Nano multiplate reader, Tecan, Männedorf, Sweden). The reference substance was EDTA at a concentration of 125 µg/mL. The metal ion chelating ability was expressed as the percentage of ion binding relative to a control sample without the extract.

#### 4.7.4. Elastase Inhibition Ability Assay

The elastase inhibition assay was performed using the method described by Wittenauer [61]. The 96-well plate was placed in a multiwell plate reader (Infinite M Nano multiplate reader, Tecan, Männedorf, Sweden) and the absorbance was measured at 405 nm. The elastase inhibition ability was expressed as the percentage reduction in elastase activity relative to a control sample without the extract.

#### 4.7.5. Tyrosinase Inhibition Ability Assay

The anti-tyrosinase activity of plant extracts was determined using an assay based on the inhibition of mushroom tyrosinase [2]. The assay was performed in a 96-well microplate format absorbance was measured at 475 nm at time intervals of 0, 1, 2, 3, 4, 5, 10, 15, 30, 45, and 60 min. Kojic acid was used as a positive control inhibitor at concentrations of 25, 50, and 100 μg/mL.

The tyrosinase inhibition activity was measured by comparing the slope of the absorbance curve of the tested sample to that of a control sample (kojic acid), with inhibition expressed as the percentage reduction in slope relative to the control.

#### 4.7.6. Anti-Microbial Assay

Microbiological tests were performed on extracts from in vitro cultures grown for 20 days as these showed the highest content of active ingredients, antioxidant potential, and elastase-inhibiting activity.

The anti-microbial activity of the tested dried extracts of *N. officinale* microshoot cultures and classical antibiotics (tetracycline) and antifungal drug (fluconazole) was assessed against Gram-positive bacteria (*S. epidermidis* ATCC 12228, *S. aureus* ATCC 29213, *C. acnes*, ATCC 6919, *C. acnes* ATCC 11827), Gram-negative bacteria (*E. coli* ATCC 25922) and fungi (*Candida albicans* ATCC 14053). Their anti-microbial activity was characterized evaluation of MIC and MBC/MFC (minimum fungicidal concentration) in the case of *C. albicans*. Dried extracts were dissolved in DMSO. Broth microdilution assays were adopted and performed in accordance with the guidelines of EUCAST [62]. Briefly, for the broth microdilution assay, 96-well microtiter plates were used. Serial 2-fold dilutions (from 10 mg/mL to 0.156 mg/mL) of the tested extract and antibiotics (256 µg/mL–0.25 µg/mL) were prepared in Mueller-Hinton Broth (Thermofisher Scientific, Waltham, USA) for *S. epidermidis*, *S. aureus*, *E. coli* strains, Brucella Broth (Thermofisher Scientific, Waltham, USA) for *C. acnes* and RPMI broth (Sigma-Aldrich, Saint Louis, USA) for *C. albicans*. Isolates of *S. epidermidis*, *S. aureus*, and *E. coli* were prepared from fresh 18–24 h cultures and incubated at 37 °C. *C. acnes*, being an anaerobic bacterium, required incubation for up to 5 days at 37 °C. In the case of *C. albicans*, the incubation lasted for 24–48 h and was performed using a solid medium at 35 °C. In the case of bacteria, the final concentration of cells was about 5 × 10^5^ CFU/mL per well, and in the case of fungi, it was about 2 × 10^7^ CFU/mL. The MIC values were read macroscopically. After the determination of the MIC, the MBC/MFC was assessed by transferring samples from the wells onto LB agar (for *S. aureus*, *S. epidermidis*, and *E. coli*) and incubating at 37 °C in aerobic conditions for another 18 ± 2 h. For *C. acnes*, the Schaedler agar was used, and the incubation was performed for 5 days in anaerobic conditions at 37 °C. For *C. albicans*, the Sabouraud agar was used, and incubation was maintained at 35 °C for 24–48 h. The MBC/MFC values were recorded as the concentrations at which no bacterial or fungi growth was observed on the solid medium.

### 4.8. Study on Cosmetic Formulation

#### 4.8.1. Preparation of Emulsion

The cosmetic face emulsion formulation with the experimental aqueous extract of *N. officinale* herb was developed. Both the face emulsion with the extract and the control emulsion (without the extract) were prepared based on the composition described in detail in Table 5.

The oil and water phases were heated separately to 70 °C in a water bath using a crystallizer filled with water and a magnetic stirrer with heating (C-MAG HS7, IKA, Warsaw, Poland) to ensure complete dissolution of the ingredients. The oil phase was additionally stirred using a mechanical stirrer (uniSTIRRER OH2, LGG Labware, Meckenheim, Germany) for better homogeneity. The water phase was then gradually added to the oil phase in small portions while being vigorously mixed with the mechanical stirrer at a speed of 600 rpm. The two phases were combined for approximately 15 min. After the phases were fully combined, the magnetic stirrer with heating was removed, and the emulsion was further mixed using the mechanical stirrer for an additional 10 min until it cooled to room temperature. Once the temperature of the emulsion dropped to 40 °C, the watercress extract was added to the mixture. The final emulsion was then transferred to a storage container.

#### 4.8.2. Physicochemical Tests

##### Stability Tests and pH Measurement

The obtained emulsions were subjected to stability tests. The first one was a centrifuge test carried out in a HETTICH EBA 20 centrifuge, set to 3500 rpm for 20 min of centrifugation.

The second stability test conducted was a temperature shock test. Each sample was transferred to a smaller screw-cap container and placed in a freezer at −18 °C for 24 h. Following this, the samples were moved to room temperature for 24 h and then transferred to an incubator set at 40 °C. This entire cycle was repeated three times to complete the test.

The pH value was measured using a Seven Multi pH meter (Mettler Toledo, Columbus, OH, USA).

##### Rheological Examination of Emulsions

The measurements of the rheological properties were performed using a rotational rheometer (R/S Plus Rheometer, Brookfield, Middlebora, MA, USA), with a C25-2 cone-plate configuration, using Rheo3000 software (https://store.brookfieldengineering.com/rheo3000-software-standard-edition/, accessed on 9 February 2025). The measurement temperature was 25 °C, the shear rate range was 1–1000 1/s, the measurement time was 60 s, and the number of measurement points was 60.

#### 4.8.3. The Total Antioxidant and Chelating Ability of the Emulsions

For the prepared emulsions, measurements of antioxidant activity and the ability to chelate iron (II) ions were conducted. For this purpose, 1 g of the tested emulsion was weighed and dissolved in ethanol (99%, Avantor Performance, Katowice, Poland) using an ultrasonic bath (10 min, 50 Hz). Then, the samples were centrifuged for 10 min at 3500 rpm. The supernatant was analyzed for total antioxidant potential using the DPPH assay according to the procedures described in Section 4.7.2. The chelating ability was assessed using a method described previously [17].

### 4.9. Statistical Analysis

All measurements for each parameter were performed in triplicate, and the results are presented as the mean value of the three measurements, along with the corresponding standard deviation, ensuring accurate and consistent processing of the experimental results RStudio, v. 2023.03.1+446 software was used in statistical analysis. All the data were expressed as mean values ± standard deviation (±SD). Pairwise comparisons were performed using Tukey’s test with a significance level of *p* < 0.05p. The charts were prepared using Inkscape v. 1.2.2.

## 5. Conclusions

The analysis of the antioxidant potential, as well as the anti-elastase, anti-tyrosinase, and anti-microbial properties, of extracts from *N. officinale* cultivated in high-yield in TIS PlantForm bioreactors, was conducted for the first time. Depending on the growth duration, differences in the GSL content were observed. The analysis of antioxidant potential showed differences depending on the extract tested (ethanol and aqueous extracts). Among the PlantForm bioreactor microshoot cultures, the highest antioxidant potential was obtained for ethanol extracts after 20 days of growth period; only in the ABTS assay did extracts from the herb exhibit higher antioxidant potential than extracts of PlantForm microshoot cultures. The assessment of anti-elastase and skin-lightening activity showed significantly stronger elastase and tyrosinase inhibition in extracts from microshoot cultures compared to those from the herb of the parent plant. In addition, extracts from 20-day microshoot cultures inhibited the growth of bacteria including *C. acnes*, *S. aureus*, *S. epidermidis*, *E. coli*, and fungi such as *C. albicans*.

Phytochemical and biological analyses of extracts from in vitro cultures, combined with the growing interest in using such cultured tissue extracts in cosmetic production prompted us to develop a cream/emulsion formulation with potential anti-aging activity. The emulsion met the requirements for products intended for skin application and demonstrated effectiveness in elastase inhibition, free radical reduction, and iron ion chelation. This is particularly significant for its potential to provide protective, rejuvenating, and safeguarding benefits for the skin.

In summary, the presented extensive studies demonstrated success in obtaining high amounts of *N. officinale* microshoots independently of the external environment in efficient, modern TIS-type bioreactors. Extracts from the obtained tissue showed effective antioxidant and anti-microbial activities and a high content of active compounds. The obtained results were competitive with extracts from the herb of the plant growing in its natural environment. The final formulation of the cream, with its potential anti-aging properties, underscores the high applicability of the obtained tissue for practical purposes.

## Figures and Tables

**Table 1:** Qualitative and quantitative (mg/100 g DW ± SD) profiles of GSL compounds detected in *N. officinale* extracts from herbs and microshoots cultivated in the PlantForm bioreactor, confirmed by UHPLC-DAD-MS/MS.

Aminoacid Precursor	Glucosinolate (GSL) (Trivial Name)	*t*_R_ (min)	[M + Na]^+^	Content		
				In Vitro Cultures		Plant Material Acc. to [29]
				Growth Cycles (Days)		
				10 Days	20 Days	
Methionine derived	6-(Methylsulfinyl)hexyl GSL (Glucohesperin)	5.20	408	n.d.	n.d.	tr
	7-(Methylsulfinyl)heptyl GSL	6.20	422	tr	tr	92.27 ± 0.48 ^a^
	8-(Methylsulfinyl)octyl GSL (Glucohirsutin)	7.20	436	tr	tr	42.79 ± 0.49 ^b^
	7-(Methylsulfanyl)heptyl GSL	10.60	406	n.d.	n.d.	tr
	8-(Methylsulfanyl)octylGSL	12.37	420	n.d.	n.d.	tr
Phenylalanine derived	2-Phenylethyl GSL (Gluconasturtiin)	7.70	366	207.91 ± 21.60	268.04 ± 33.45	640.94 ± 2.96 ^c^
Tryptophan derived	Indol-3-ylmethyl GSL (Glucobrassicin)	7.00	391	493.00 ± 0.45	n.d.	tr
	4-Methoxyindol-3-ylmethyl GSL (4-Methoxyglucobrassicin)	7.80	421	n.d.	n.d.	23.47 ± 0.96 ^d^

[M + Na]^+^, sodium adduct of desulfoglucosinolate; DW, dry weight; tr < 0.1 μmol/g DW; n.d., not detected. Data are expressed as mean values with standard deviations (±SD) and resulted from three independent experiments with three repetitions (*n* = 3); a significant difference is marked with small letters (*p* < 0.05).

**Table 2:** Total polyphenol content (TPC) (mg GAE/100 g DW ± SD) and quantities of the estimated phenolic compounds (mg/100 g DW±SD) in *N. officinale* extracts from herb and microshoots cultivated in PlantForm bioreactor.

Polyphenols	Contents		
	Growth Period (Days)		Plant Material Acc. to [29]
	10 Days	20 Days	
TPC	2540 ± 160 ^a^	2690 ± 240 ^a^	2940 ± 80 ^b^
*p*-Coumaric acid	n.d.	n.d.	3.10 ± 0.21
Gallic acid	n.d.	n.d.	26.55 ± 4.72
Ferulic acid	57.99 ± 4.87 ^c^	87.78 ± 8.81 ^d^	12.69 ± 1.13 ^e^
Protocatechuic acid	8.75 ± 0.76 ^f^	15.65 ± 1.46 ^f^	196.11 ± 18.23 ^h^
Sinapinic acid	114.83 ± 10.92 ^i^	78.65 ± 6.99 ^j^	n.d.
Isoquercitrin	n.d.	n.d.	57.05 ± 5.11
Kaempferol *O*-rhamnohexoside	n.d.	n.d.	0.18 ± 0.02
Rutoside	13.40 ± 1.28 ^k^	33.27 ± 3.03 ^l^	7.20 ± 0.67 ^m^

n.d.—not detected; the values are presented as mean values with standard deviations (±SD) and resulted from three independent experiments with three repetitions (*n* = 9); a significant difference is marked with small letters (*p* < 0.05).

**Table 3:** Anti-microbial activity of extracts from *N. officinale* microshoot cultures grown in PlantForm bioreactors over a 20-day growth period.

Microorganism			MIC (mg/mL)	MBC or MFC (mg/mL)	Reference Drug	MIC (µg/mL)	MBC or MFC (µg/mL)
Bacteria	Gram-positive	*Staphylococcus epidermidis* ATCC 12228	1.25 ± 0.01	2.5	Tetracycline	128 ± 0.10	256
		*Staphylococcus aureus* ATCC 29213	1.25 ± 0.03	5	Tetracycline	1 ± 0.06	0.5
		*Cutibacterium acnes* ATCC 11827	0.625 ± 0.01	0.625	Tetracycline	0.5 ± 0.02	16
		*Cutibacterium acnes* ATCC 6919	1.25 ± 0.05	1.25	Tetracycline	0.5 ± 0.01	16
	Gram-negative	*Escherichia coli* ATCC 25922	1.25 ± 0.03	2.5	Tetracycline	0.25 ± 0.02	0.25
Fungi		*Candida albicans* ATCC 14053	1.25 ± 0.04	1.25	Fluconazole	1 ± 0.05	0.5

**Table 4:** Emulsion viscosity values for selected shear rates.

Emulsion	Viscosity at Shear Rate 50 1/s [Pa]	Viscosity at Shear Rate 100 1/s [Pa]
Face emulsion with *N. officinale* extract	1.8746 ± 0.10	0.9215 ± 0.10
Control emulsion	1.0709 ± 0.20	0.5763 ± 0.06

**Table 5:** Ingredients and their composition used for preparation of experimental face emulsions.

Phase	Control Emulsion		Face Emulsion with *N. officinale* Extract	
	Ingredient	Concentration %	Ingredient	Concentration %
Oil phase	Lanolin	9	Lanolin	9
	Cera flava	6	Cera flava	6
	Shea butter	7	Shea butter	7
	Rosehip seed oil	40	Rosehip seed oil	40
Water phase	D-Panthenol 75%	3	D-panthenol 75%	3
	Glycerine	3.2	Glycerine	3.2
	Rose hydrolate	15	Rose hydrolate	15
	Deionized water	16.2	Deionized water	14.2
	FEOG	0.6	FEOG	0.6
	Aqueous extract of *N. officinale* herb	-	Aqueous extract of *N. officinale* herb	2

## Data Availability

Data are contained within the article and Supplementary Materials.

## Supplementary Materials

The following supporting information can be downloaded at https://www.mdpi.com/article/10.3390/molecules30040936/s1, Figure S1: Morphological appearance of *N. officinale* microshoot cultures cultivated in bioreactor PlantForm after 10 (A) and 20 days (B) of growth period; and parent plant (C). Ref. [63] is cited in the Supplementary Materials.

## References

[1] Patel M.K., Pandey S., Kumar M., Haque I., Pal S. (2021). Plants Metabolome Study: Emerging Tools and Techniques. Plants.

[2] Malinowska M.A., Billet K., Drouet S., Munsch T., Unlubayir M., Tungmunnithum D., Giglioli-Guivarc’H N., Hano C., Lanoue A. (2020). Grape Cane Extracts as Multifunctional Rejuvenating Cosmetic Ingredient: Evaluation of Sirtuin Activity, Tyrosinase Inhibition and Bioavailability Potential. Molecules.

[3] Del-Castillo-Alonso M.Á., Diago M.P., Tomás-Las-Heras R., Monforte L., Soriano G., Martínez-Abaigar J., Núñez-Olivera E. (2016). Effects of Ambient Solar UV Radiation on Grapevine Leaf Physiology and Berry Phenolic Composition along One Entire Season under Mediterranean Field Conditions. Plant Physiol. Biochem..

[4] Marchev A.S., Georgiev M.I. (2020). Plant in Vitro Systems as a Sustainable Source of Active Ingredients for Cosmeceutical Application. Molecules.

[5] Flieger J., Raszewska-Famielec M., Radzikowska-Büchner E., Flieger W. (2024). Skin Protection by Carotenoid Pigments. Int. J. Mol. Sci..

[6] Nasirmahalleh N.M., Hemmati M., Ghorbani F. (2025). Anti-Oxidant and Anti-Inflammatory Action of Calorie Restriction and Quercetin in Two Age Groups of Rats: Involvement of Thioredoxin and Heme. Hum. Gene.

[7] Choi J.K., Kim S.H. (2013). Rutin Suppresses Atopic Dermatitis and Allergic Contact Dermatitis. Exp. Biol. Med..

[8] Patil S.L., Mallaiah S.H., Patil R.K. (2013). Antioxidative and Radioprotective Potential of Rutin and Quercetin in Swiss Albino Mice Exposed to Gamma Radiation. J. Med. Phys..

[9] Zhu H., Xu Z., Zheng R., Kang J., Gao L., Xiong S., Liu Y. (2025). Discovering the Antioxidant Properties and Interaction Mechanisms of Rosmarinic Acid and Kaempferol with Grass Carp Hemoglobin: An Experimental Studies and Molecular Simulations Perspective. Food Chem..

[10] Xia Y., Zhang H., Wu X., Xu Y., Tan Q. (2024). Resveratrol Activates Autophagy and Protects from UVA-Induced Photoaging in Human Skin Fibroblasts and the Skin of Male Mice by Regulating the AMPK Pathway. Biogerontology.

[11] Deepika, Maurya P.K. (2023). Protective Effect of Ellagic Acid on Erythrocytes Subjected to Oxidative Stress during Human Ageing. Indian J. Nat. Prod. Resour..

[12] Al-Shabib N.A., Husain F.M., Ahmad I., Khan M.S., Khan R.A., Khan J.M. (2017). Rutin Inhibits Mono and Multi-Species Biofilm Formation by Foodborne Drug Resistant Escherichia Coli and Staphylococcus Aureus. Food Control.

[13] Ming D., Wang D., Cao F., Xiang H., Mu D., Cao J., Li B., Zhong L., Dong X., Zhong X. (2017). Kaempferol Inhibits the Primary Attachment Phase of Biofilm Formation in *Staphylococcus aureus*. Front. Microbiol..

[14] Franco F.N., Peixoto B.E., de Araújo G.R., Chaves M.M. (2025). Silencing of the Nrf2 Pathway in Aging Promotes a Decrease in the Anti-Inflammatory Effect of Resveratrol. Arch. Gerontol. Geriatr..

[15] Özer Ö., Mutlu B., Kivçak B. (2007). Antityrosinase Activity of Some Plant Extracts and Formulations Containing Ellagic Acid. Pharm. Biol..

[16] Zhao L., Jin L., Yang B. (2023). Protocatechuic Acid Inhibits LPS-Induced Mastitis in Mice through Activating the Pregnane X Receptor. J. Cell. Mol. Med..

[17] Sharafan M., Malinowska M.A., Kubicz M., Kubica P., Marin-Pierre G., Abdallah C., Ferrier M., Hano C., Giglioli-Guivarc N., Sikora E. (2023). Shoot Cultures of *Vitis vinifera* (Vine Grape) Different Cultivars as a Promising Innovative Cosmetic Raw Material—Phytochemical Profiling, Antioxidant Potential, and Whitening Activity. Molecules.

[18] Hagaggi N.S.A., Abdul-Raouf U.M., Radwan T.A.A. (2024). Variation of Antibacterial and Antioxidant Secondary Metabolites and Volatiles in Leaf and Callus Extracts of Phulai (*Acacia modesta* Wall.). BMC Plant Biol..

[19] Jesionek A., Kokotkiewicz A., Krolicka A., Zabiegala B., Luczkiewicz M. (2018). Elicitation Strategies for the Improvement of Essential Oil Content in *Rhododendron tomentosum* (Ledum Palustre) Bioreactor-Grown Microshoots. Ind. Crops Prod..

[20] Satdive R., Shinde A.N., Singh S., Kamble S., Singh S., Malpathak N., Fulzele D.P. (2015). Aggregate Cell Suspension Cultures of Psoralea Corylifolia Improved Phytoestrogens Production. Biotechnol. Bioprocess Eng..

[21] Swamy M.K., Nath S., Paul S., Jha N.K., Purushotham B., Rohit K.C., Dey A. (2021). Biotechnology of Camptothecin Production in Nothapodytes Nimmoniana, *Ophiorrhiza* Sp. and *Camptotheca acuminata*. Appl. Microbiol. Biotechnol..

[22] Szopa A., Kubica P., Ekiert H. (2018). Agitated Shoot Cultures of *Aronia arbutifolia* and *Aronia × prunifolia*: Biotechnological Studies on the Accumulation of Phenolic Compounds and Biotransformation Capability. Plant Cell. Tissue Organ Cult..

[23] IUCN The IUCN Red List of Threatened Species. https://www.iucnredlist.org/.

[24] Boligon A.A., Janovik V., Boligon A.A., Pivetta C.R., Pereira R.P., Da Rocha J.B.T., Athayde M.L. (2013). HPLC Analysis of Polyphenolic Compounds and Antioxidant Activity in *Nasturtium officinale*. Int. J. Food Prop..

[25] Martínez-Sánchez A., Gil-Izquierdo A., Gil M.I., Ferreres F. (2008). A Comparative Study of Flavonoid Compounds, Vitamin C, and Antioxidant Properties of Baby Leaf Brassicaceae Species. J. Agric. Food Chem..

[26] Afsharypuor S., Salehi M. (2008). Volatile Constituents of Leaves and Stems of *Nasturtium officinale* R. Br. J. Essent. Oil Res..

[27] Jeon J., Bong S.J., Park J.S., Park Y.K., Arasu M.V., Al-Dhabi N.A., Park S.U. (2017). De Novo Transcriptome Analysis and Glucosinolate Profiling in Watercress (*Nasturtium officinale* R. Br.). BMC Genom..

[28] Klimek-Szczykutowicz M., Szopa A., Dziurka M., Komsta Ł., Tomczyk M., Ekiert H. (2020). The Influence of *Nasturtium officinale* R. Br. Agar and Agitated Microshoot Culture Media on Glucosinolate and Phenolic Acid Production, and Antioxidant Activity. Biomolecules.

[29] Klimek-Szczykutowicz M., Dziurka M., Blažević I., Đulović A., Granica S., Korona-Glowniak I., Ekiert H., Szopa A. (2020). Phytochemical and Biological Activity Studies on *Nasturtium officinale* (Watercress) Microshoot Cultures Grown in RITA^®^ Temporary Immersion Systems. Molecules.

[30] Klimek-Szczykutowicz M., Dziurka M., Blažević I., Đulović A., Miazga-Karska M., Klimek K., Ekiert H., Szopa A. (2021). Precursor-Boosted Production of Metabolites in *Nasturtium officinale* Microshoots Grown in Plantform Bioreactors, and Antioxidant and Antimicrobial Activities of Biomass Extracts. Molecules.

[31] Klimek-Szczykutowicz M., Dziurka M., Blažević I., Đulović A., Apola A., Ekiert H., Szopa A. (2022). Impacts of Elicitors on Metabolite Production and on Antioxidant Potential and Tyrosinase Inhibition in Watercress Microshoot Cultures. Appl. Microbiol. Biotechnol..

[32] Sikora E., Miastkowska M., Lasoń E. (2020). Selected Skin Delivery Systems.

[33] Giallourou N., Oruna-Concha M.J., Harbourne N. (2016). Effects of Domestic Processing Methods on the Phytochemical Content of Watercress (*Nasturtium officinale*). Food Chem..

[34] Rubin E., Aziz Z.A., Surugau N. (2018). Glucosinolates Content of in Vitro Grown *Nasturtium officinale* (Watercress). ASM Sci. J..

[35] Jafernik K., Kubica P., Dziurka M., Kulinowski Ł., Korona-głowniak I., Elansary H.O., Walig P., Skalicka-wo K., Szopa A. (2024). Comparative Assessment of Lignan Profiling and Biological Activities of *Schisandra henryi* Leaf and In Vitro PlantForm Bioreactor-Grown Culture Extracts. Pharmaceuticals.

[36] Airò M., Mammano M.M., Giardina G., Giovino A. (2017). Temporary Immersion System: An Efficient Technique to Improve the Plumeria Rubra L. Scale-Up. Acta Hortic..

[37] Makowski W., Królicka A., Tokarz B., Szopa A., Ekiert H., Tokarz K.M. (2023). Temporary Immersion Bioreactors as a Useful Tool for Obtaining High Productivity of Phenolic Compounds with Strong Antioxidant Properties from *Pontechium maculatum*. Plant Cell. Tissue Organ Cult..

[38] Arena D., Ben Ammar H., Major N., Kovačević T.K., Goreta Ban S., Treccarichi S., Lo Scalzo R., Branca F. (2024). Light Use Efficiency of Broccoli (*Brassica oleracea* var. *italica* Plenck) and Rocket (*Eruca sativa* L.) during the Initial Plant Growth Stages. Sci. Hortic..

[39] Al-Ogaili N. (2024). The Evaluation of Total Flavonoids, Total Phenolic Content and Biological Activity of Iraqi *Lipedium sativum* L. Crude Extract Obtained by Optimized Ultrasound Assisted Extraction Conditions. Plant Sci. Today.

[40] Arena P., Miceli N., Marino A., Davì F., Cavò E., Spadaro V., Raimondo F.M., Cacciola F., Laganà Vinci R., Mondello L. (2023). Comparative Study on Phenolic Profile and Biological Activities of the Aerial Parts of *Sinapis pubescens* L. Subsp. Pubescens (Brassicaceae) Wild from Sicily (Italy). Chem. Biodivers..

[41] Mansinhos I., Gonçalves S., Rodríguez-Solana R., Pereira-Caro G., Moreno-Rojas J., Romano A. (2024). Nutrient Deficiency-Induced Stress Improves Skincare Effects and Phytochemical Content of Green Extracts from Lamiaceae In Vitro Cultures. Horticulturae.

[42] Bose B., Choudhury H., Tandon P., Kumaria S. (2017). Studies on Secondary Metabolite Profiling, Anti-Inflammatory Potential, in Vitro Photoprotective and Skin-Aging Related Enzyme Inhibitory Activities of *Malaxis acuminata*, a Threatened Orchid of Nutraceutical Importance. J. Photochem. Photobiol. B Biol..

[43] Szewczyk K., Pietrzak W., Klimek K., Miazga-Karska M., Firlej A., Flisiński M., Grzywa-Celińska A. (2021). Flavonoid and Phenolic Acids Content and in Vitro Study of the Potential Anti-Aging Properties of *Eutrema japonicum* (Miq.) Koidz Cultivated in Wasabi Farm Poland. Int. J. Mol. Sci..

[44] Zafar R., Zahoor M., Shah A.B., Majid F. (2017). Determination of Antioxidants and Antibacterial Activities, Total Phenolic, Polyphenol and Pigment Contents in *Nasturtium officinale*. Pharmacologyonline.

[45] Quezada-Lázaro R., Fernández-Zuñiga E.A., García A., Garza-González E., Alvarez L., Camacho-Corona M.R. (2016). Antimycobacterial Compounds from *Nasturtium officinale*. Afr. Tradit. Complement. Altern. Med..

[46] Akaza N., Takasaki K., Matsudaira T., Usui A., Iijima A., Miura S., Yashiro Y. (2023). Relationship between Skin Fungal and Bacterial Microbiomes and Skin PH. Int. J. Cosmet. Sci..

[47] Ordoñez-Toro A., Montero-Vilchez T., Muñoz-Baeza J., Sanabria-De-la-Torre R., Buendia-Eisman A., Arias-Santiago S. (2022). The Assessment of Skin Homeostasis Changes after Using Different Types of Excipients in Healthy Individuals. Int. J. Environ. Res. Public Health.

[48] Pinto D., Lameirão F., Delerue-Matos C., Rodrigues F., Costa P. (2021). Characterization and Stability of a Formulation Containing Antioxidants-Enriched *Castanea sativa* Shells Extract. Cosmetics.

[49] Klimek-Szczykutowicz M., Szopa A., Blicharska E., Dziurka M., Komsta Ł., Ekiert H. (2019). Bioaccumulation of Selected Macro- and Microelements and Their Impact on Antioxidant Properties and Accumulation of Glucosinolates and Phenolic Acids in in Vitro Cultures of *Nasturtium officinale* (Watercress) Microshoots. Food Chem..

[50] Murashige T., Skoog F. (1962). A Revised Medium for Rapid Growth and Bioassays with Tobacco Tissue Cultures. Physiol. Plant..

[51] Blažević I., Đulović A., Čikeš Čulić V., Burčul F., Ljubenkov I., Ruščić M., Generalić Mekinić I. (2019). *Bunias erucago* L.: Glucosinolate Profile and in Vitro Biological Potential. Molecules.

[52] Grosser K., van Dam N.M. (2017). A Straightforward Method for Glucosinolate Extraction and Analysis with High-Pressure Liquid Chromatography (HPLC). J. Vis. Exp..

[53] Brown P.D., Tokuhisa J.G., Reichelt M., Gershenzon J. (2003). Variation of Glucosinolate Accumulation among Different Organs and Developmental Stages of *Arabidopsis thaliana*. Phytochemistry.

[54] Wathelet J.P., Iori R., Leoni O., Quinsac O., Palmieri S. (2004). Guidelines for Glucosinolate Analysis in Green Tissues Used for Biofumigation. Agroindustria.

[55] Singleton V., Rossi J. (1965). Colorimetry of Total Phenolics with Phosphomolybdic-Phosphotungstic Acid Reagents. Am. J. Enol. Vitic..

[56] Sułkowska-Ziaja K., Maślanka A., Szewczyk A., Muszyńska B. (2017). Physiologically Active Compounds in Four Species of Genus *Phellinus*. Nat. Prod. Commun..

[57] Ellnain-Wojtaszek M., Zgórka G. (1999). High-Performance Liquid Chromatography and Thin-Layer Chromatography of Phenolic Acids from *Gingko biloba* L. Leaves Collected within Vegetative Period. J. Liq. Chromatogr. Relat. Technol..

[58] Re R., Pellegrini N., Proteggente A., Pannala A., Yang M., Rice-Evans C. (1999). Antioxidant Activity Applying an Improved ABTS Radical Cation Decolorization Assay. Free Radic. Biol. Med..

[59] Brand-Williams W., Cuvelier M., Berset C. (1995). Use of a Free Radical Method to Evaluate Antioxidant Activity. LWT-Food Sci. Technol..

[60] Adjimani J.P., Asare P. (2015). Antioxidant and Free Radical Scavenging Activity of Iron Chelators. Toxicol. Rep..

[61] Wittenauer J., Sonja M., Sußmann D., Schweiggert-Weisz U., Carle R. (2015). Inhibitory Effects of Polyphenols from Grape Pomace Extract on Collagenase and Elastase Activity. Fitoterapia.

[62] EUCAST. https://www.eucast.org.

[63] Grzegorczyk I., Wysokińska H. (2008). Liquid Shoot Culture of *Salvia officinalis* L. for Micropropagation and Production of Antioxidant Compounds; Effects of Triacontanol. Acta Soc. Bot. Pol..

