# Supplementary material for: Nasturtium officinale Microshoot Culture Multiplied in PlantForm Bioreactor—Phytochemical Profiling and Biological Activity
Source: Molecules. 2025 Feb 18;30(4):936. doi: 10.3390/molecules30040936 (PMC11858548; doi:10.3390/molecules30040936)
Supplement: Supplementary file 1 [file molecules-30-00936-s001.zip › molecules-3359838-supplementary.pdf]

Supplementary Data

Nasturtium officinale Microshoot Culture Multiplied in PlantForm Bioreactor—Phytochemical Profiling and Biological Activity

Marta Klimek-Szczykutowicz [1,*], Magdalena Anna Malinowska [2], Aleksandra Gałka [2], Ivica Blažević [3], Azra Đulović [3], Paulina Paprocka [4], Małgorzata Wrzosek [1,5,*] and Agnieszka Szopa [6]

1 Department of Pharmaceutical Sciences, Collegium Medicum, Jan Kochanowski University in Kielce, IX Wieków Kielc 19a, 25-516 Kielce, Poland
2 Department of Organic Chemistry and Technology, Faculty of Chemical Engineering and Technology, Cracow University of Technology, Warszawska 24, 31-155 Kraków, Poland; magdalena.malinowska@pk.edu.pl (M.A.M.); aleksandra.galka01@gmail.com (A.G.)
3 Department of Organic Chemistry, Faculty of Chemistry and Technology, University of Split, Ruđera Boškovića 35, 21000 Split, Croatia; ivica.blazevic@ktf-split.hr (I.B.); azra.dulovic@ktf-split.hr (A.Đ.)
4 Department of Microbiology and Immunology, Collegium Medicum, Institute of Medical Science, Jan Kochanowski University in Kielce, IX Wiekow Kielc 19A, 25-317, Kielce, Poland; paulina.paprocka@ujk.edu.pl
5 Department of Biochemistry and Pharmacogenomics, Medical University of Warsaw, Banacha 1, 02-097 Warsaw, Poland
6 Department of Medicinal Plant and Mushroom Biotechnology, Faculty of Pharmacy, Jagiellonian University Medical College, Medyczna 9, 30-688 Kraków, Poland; a.szopa@uj.edu.pl
* Correspondence: marta.klimek-szczykutowicz@ujk.edu.pl (M.K.-S.); malgorzata.wrzosek@wum.edu.pl (M.W.)

1. *Microshoot Appearance and Biomass Growth*

The appearance of the tested bioreactor cultures was dependent on the growth period's duration (Figure 1A,B). After 10 and 20 days, *N. officinale* microshoot cultures were characterized by a green color and a large number of shoots.

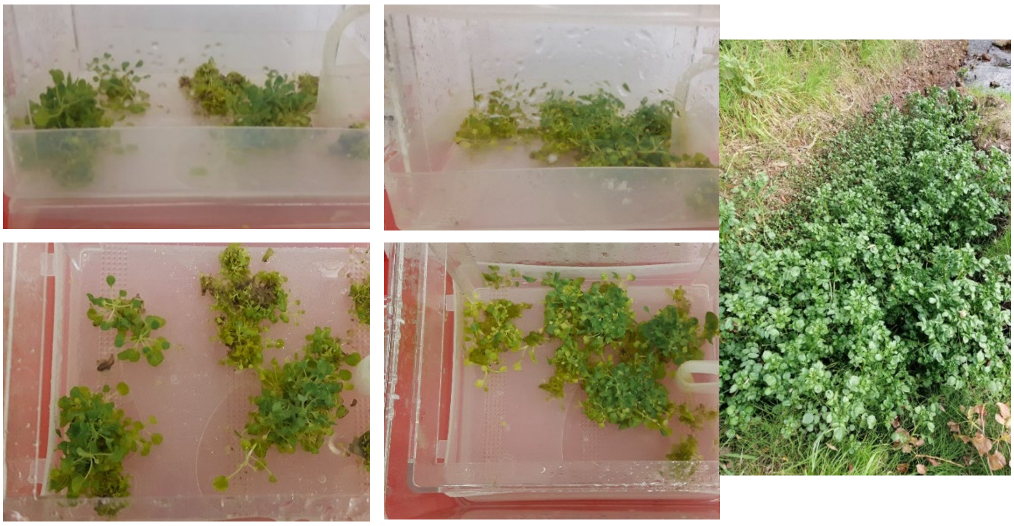

Figure S1. Morphological appearance of *N. officinale* microshoot cultures cultivated in bioreactor PlantForm after 10 (A) and 20 days (B) of growth period; and parent plant (C).

Biomass increments were measured by weighing the dry biomass obtained after 10 and 20 days of the growth period after lyophilization. For dry biomass, the Gi was calculated according to the formula: Gi = $\frac{(Dw_1 - Dw_0)}{Dw_0}$, where Dw_1 is the dry weight of microshoots observed at the end of the experiment, and Dw_0 is the dry weight of the inoculum [1].

High growth index (Gi) values were obtained for the dry biomass. Depending on the culture duration, the increases in dry biomass, expressed by Gi, ranged from 20.13 (after 10 days) up to 31.36 (after 20 days). The Gi estimated for the cultures grown over 20 days was 1.6 times higher than the increase recorded after 10 days.

References

1. Grzegorczyk, I.; Wysokińska, H. Liquid Shoot Culture of *Salvia officinalis* L. for Micropropagation and Production of Antioxidant Compounds; Effects of Triacontanol. *Acta Soc. Bot. Pol.* **2008**, *77*, 99–104, doi:https ://doi.org/10.5586/asbp.2008.013.
